# Do Arabinogalactan Proteins Occur in the Transfer Cells of *Utricularia dichotoma*?

**DOI:** 10.3390/ijms25126623

**Published:** 2024-06-16

**Authors:** Bartosz J. Płachno, Małgorzata Kapusta, Piotr Stolarczyk, Marcin Feldo, Piotr Świątek

**Affiliations:** 1 Department of Plant Cytology and Embryology, Institute of Botany, Faculty of Biology, Jagiellonian University in Kraków, 9 Gronostajowa St., 30-387 Kraków, Poland; 2 Bioimaging Laboratory, Faculty of Biology, University of Gdańsk, 59 Wita Stwosza St., 80-308 Gdańsk, Poland; malgorzata.kapusta@ug.edu.pl; 3 Department of Botany, Physiology and Plant Protection, Faculty of Biotechnology and Horticulture, University of Agriculture in Kraków, 29 Listopada 54 Ave., 31-425 Kraków, Poland; piotr.stolarczyk@urk.edu.pl; 4 Department of Vascular Surgery and Angiology, Medical University of Lublin, 16 Staszica St., 20-081 Lublin, Poland; martinf@interia.pl; 5 Institute of Biology, Biotechnology and Environmental Protection, Faculty of Natural Sciences, University of Silesia in Katowice, 9 Bankowa St., 40-007 Katowice, Poland; piotr.swiatek@us.edu.pl

**Keywords:** arabinogalactan proteins, bladderworts, carnivorous plants, cell wall, cell wall microdomains, cuticle, digestive trichomes, glands, Lentibulariaceae, transfer cells, scanning transmission electron microscopy

## Abstract

Species in the genus *Utricularia* are carnivorous plants that prey on invertebrates using traps of leaf origin. The traps are equipped with numerous different glandular trichomes. Trichomes (quadrifids) produce digestive enzymes and absorb the products of prey digestion. The main aim of this study was to determine whether arabinogalactan proteins (AGPs) occur in the cell wall ingrowths in the quadrifid cells. Antibodies (JIM8, JIM13, JIM14, MAC207, and JIM4) that act against various groups of AGPs were used. AGP localization was determined using immunohistochemistry techniques and immunogold labeling. AGPs localized with the JIM13, JIM8, and JIM14 epitopes occurred in wall ingrowths of the pedestal cell, which may be related to the fact that AGPs regulate the formation of wall ingrowths but also, due to the patterning of the cell wall structure, affect symplastic transport. The presence of AGPs in the cell wall of terminal cells may be related to the presence of wall ingrowths, but processes also involve vesicle trafficking and membrane recycling, in which these proteins participate.

## 1. Introduction

**Figure 1:**
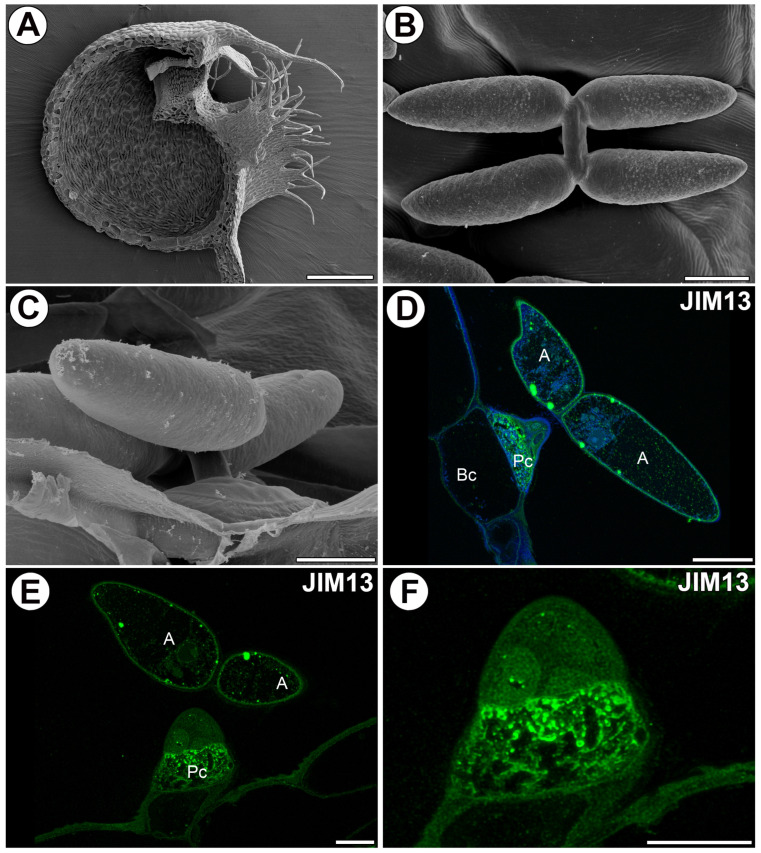
Distribution and morphology of the quadrifids of *Utricularia dichotoma* subsp. *novae-zelandiae*. Immunogold labeling of quadrifid cells with JIM13. The arabinogalactan proteins detected in the quadrifids (green color—signal of antibody). (**A**) A sagittally halved trap showing the quadrifids. Bar 500 µm. (**B**,**C**) The morphology of the quadrifids. Bars 20 µm. (**D**,**E**) Arabinogalactan proteins (labeled with JIM13) were detected in the quadrifid: arm (A), pedestal cell (Pc), basal cell (Bc). Bars 10 µm. (**F**) Strong fluorescence signal of AGP epitope (labeled with JIM13) in wall ingrowths in pedestal cell. Bar 10 µm.

*Utricularia* are carnivorous plants with traps. They are the fastest moving among the carnivorous plants [1,2] and capture fine organisms such as invertebrates, protozoa, and algae. An *Utricularia* trap is a small hollow vesicle filled with trap fluid (a bladder with elastic walls and a mobile trap door), which is called a suction trap (Figure 1A). *Utricularia* traps have extremely varied structures, often linked to taxonomic relationships [3]. The differences are mainly in the architecture of the trap entrance, the door structure, the presence of various trichomes and accessory structures, and sometimes also the thickness of the trap wall (e.g., [4,5,6,7,8]). In many *Utricularia* species, the inner trap surface is lined with glandular trichomes called quadrifids (Figure 1B,C) [9,10], the primary function of which is the secretion of digestive enzymes and resorption of released nutrients from prey, but they likely also participate in pumping out water from the trap [10,11,12,13]. Fineran and Lee [11] studied the ultrastructure of *Utricularia* quadrifids in detail and showed that the pedestal cell of this trichome is a transfer cell (sensu Pate and Gunning [14]) with an elaborate cell wall labyrinth. The terminal cells of quadrifids are highly specialized in parts (a basal part, a stalk, and an arm) with distinct structures and functions. Thus, these trichomes are excellent models for studying the specialization of cell walls (microdomains). 

Arabinogalactan proteins (AGPs) are hydroxyproline-rich glycoproteins that link the cell wall and the cytoskeleton and affect cell wall structure and symplast transport, thus playing essential roles in plants. By affecting the cytoskeleton, cell membrane, and cell wall, they have an impact on exocytosis, endocytosis, cell wall formation, and the cortical endoplasmic reticulum. They participate as regulatory or signal molecules in plant growth and developmental processes, in response to biotic and abiotic stress, and in plant sexual reproduction (e.g., [15,16,17,18,19,20,21,22,23,24]). AGPs were recorded in the wall labyrinth in transfer cells of various species (e.g., [25,26,27,28]). Płachno and Kapusta [29] localized the AGPs in quadrifids using whole-mount immunolabeled *Utricularia* traps. However, immunolabeling was only successful in the cell walls of parts of quadrifid arms, where the cuticle has an open structure. Thus, the method did not detect AGPs in the cell wall labyrinth in the pedestal cell. The main aim of this study was to determine whether AGPs occur in the cell wall ingrowths in the quadrifid cells, especially the pedestal cell. We believe that AGPs should be present in the pedestal cell for several reasons: 1. due to the presence of the wall labyrinth; 2. the high activity of this cell; and 3. the intense transport that occurs through this cell. In addition, we have found AGPs in transfer cells in the trap glands of in other carnivorous plants.

## 2. Results

### AGP Distribution in Quadrifids

The epitope recognized by the JIM13 antibody was detected in the cell walls of all quadrifid cells. However, the strong fluorescence signal detected by JIM13 was observed in the cell wall ingrowths of the pedestal cell (Figure 1D,F). A fluorescence signal detected by JIM13 was well observed in the cell walls of the arms of terminal cells. The signal occurred mainly in the inner part of the cell walls (Figure 1D,E). 

**Figure 2:**
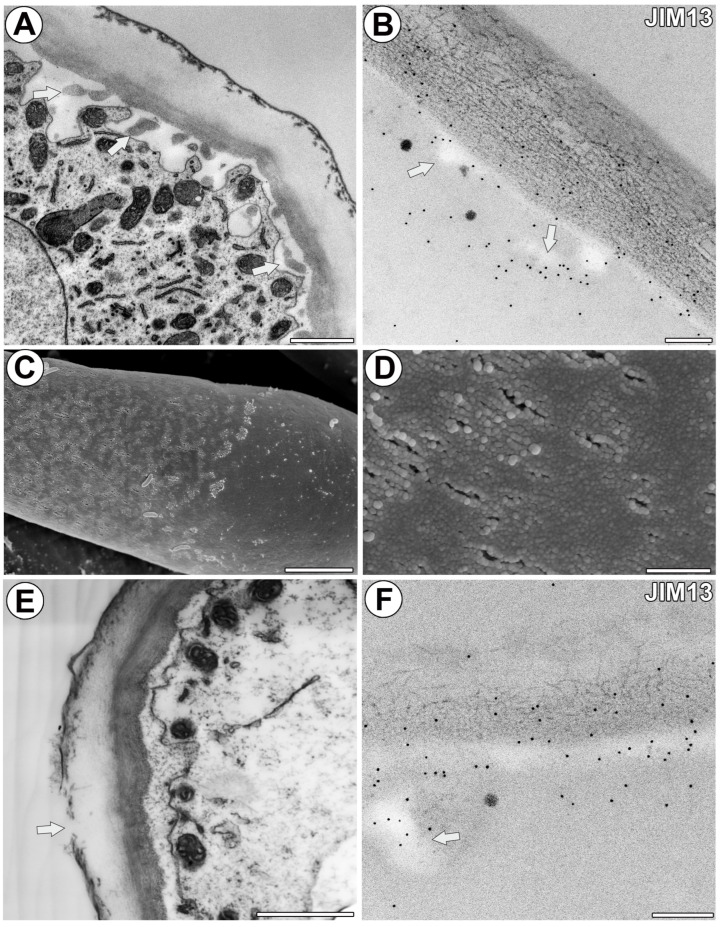
Structure of arms and the arabinogalactan proteins that were detected in the arms. (**A**) Ultrastructure of the proximal region of the arm; wall ingrowths (arrow). Bar 1 µm. (**B**) Immunogold labeling of wall ingrowths and cell wall with JIM13 in the proximal region of the arm; wall ingrowths (arrow). Bar 200 nm. (**C**,**D**) SEM photos showing cuticular discontinuities in the arms. Bars 5 µm. (**E**) Ultrastructure of the middle region of the arm; cuticular discontinuity (arrow). Bar 1 µm. (**F**) Immunogold labeling of wall ingrowths and cell wall with JIM13 in the middle region of the arm; wall ingrowths (arrow). Bar 200 nm.

**Figure 3:**
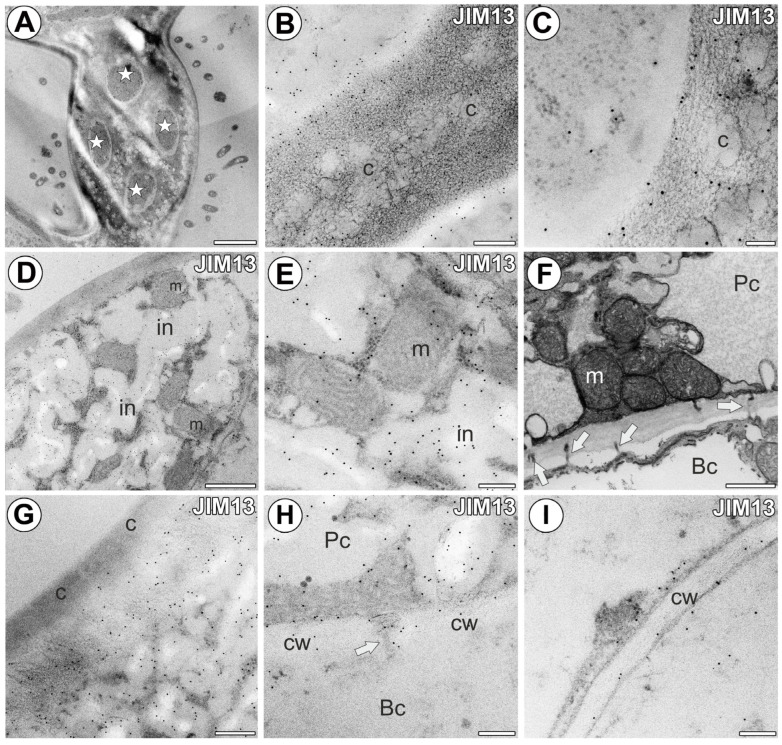
Immunogold labeling of cells with JIM13 in the quadrifid. (**A**) A section through the stalk. Note four protoplasts of the terminal cells (indicated by stars). Bar 2 µm. (**B**,**C**) Immunogold labeling with JIM13 in the stalk. Note that gold particles occur mostly in the non-cutinized cell wall parts; cutin cystoliths (c). Bar 300 nm and bar 100 nm. (**D**,**E**) Immunogold labeling with JIM13 in the pedestal cell; wall ingrowths (in), mitochondrion (m). Bar 800 nm and bar 200 nm. (**F**) Ultrastructure of the pedestal cell (Pc) and basal cell (Bc). Note numerous mitochondria (m) and plasmodesmata (arrow). Bar 600 nm. (**G**) Immunogold labeling with JIM13 in the pedestal cell. Note that gold particles occur mostly in the non-cutinized cell wall parts; cutinized cell wall part (c). Bar 300 nm. (**H**) Immunogold labeling with JIM13 in the border between pedestal cell (Pc) and basal cell (Bc). Note gold particles near plasmodesma (arrow); cell wall (cw). Bar 200 nm. (**I**) Immunogold labeling with JIM13 in the border between parenchyma cell and basal cell; cell wall (cw). Bar 200 nm.

In the proximal region of the arm of the terminal cell, where there was a nucleus and numerous organelles, the outer part of the cell wall was impregnated by cutin, and a cuticle occurred (Figure 2A). In the proximal region of the arm of the terminal cell, the immunogold labeling with JIM13 showed that the AGP epitopes were localized in the inner part of the cell wall and cell wall ingrowths (Figure 2B). In the middle and distal regions of the arm, the cuticle had discontinuities (cuticular pores), which were observable using SEM (Figure 2C,D) and TEM (Figure 2E). In these regions, gold particles were mainly distributed in the inner part of the cell wall and also in the cell wall ingrowths (Figure 2F). The cell wall ingrowths were simple or branched (reticulated type). The gold particles were also found in the cell wall where the terminal cells joined, forming a quadrifid stalk (Figure 3A–C). Here, the wall of the stalk was densely impregnated by cutin, and cutin cystoliths occurred (Figure 3B,C). The gold particles occurred in fibrillar, unimpregnated wall material in the stalk; however, some gold particles also occurred in the impregnated cell wall (Figure 3C).

The immunogold labeling with JIM13 showed that the AGP epitopes were localized in the cell walls and wall ingrowths of the pedestal cells. The gold particles were particularly numerous in the cell wall ingrowths (Figure 3D,E). Some gold particles also occurred in the cytoplasm. In the pedestal cells, there were numerous well-differentiated mitochondria, which are dominated organelles (Figure 3F). The proximal region of the lateral cell wall was completely impregnated by cutin material. The distal region of the lateral cell wall was partially impregnated by cutin material. The gold particles were located in the regions of the cell wall not impregnated by cutin material (Figure 3G). The gold particles were also found in the transverse wall between the pedestal cell and the basal epidermal cell, near to the plasmodesmata (Figure 3H). The immunogold labeling with JIM13 also showed that the AGP epitopes were localized in the cell walls of basal cells (Figure 3I).

**Figure 4:**
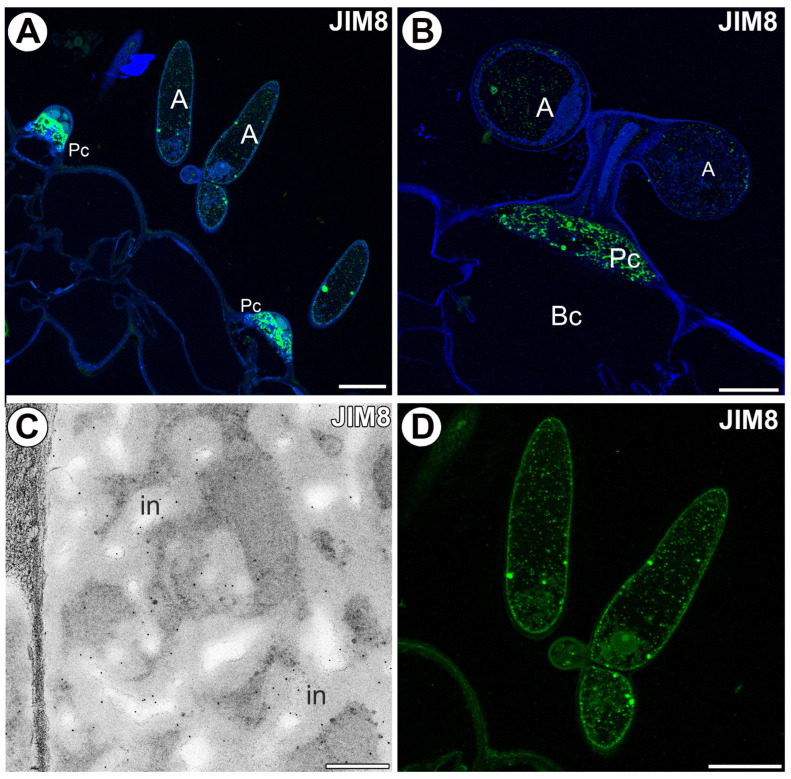
Arabinogalactan proteins detected in the quadrifids labeled with JIM8 (green color—signal of antibody). (**A**,**B**) Arabinogalactan proteins (labeled with JIM8) were detected in the quadrifid. Note the strong fluorescence signal in the pedestal cell; arm (A), pedestal cell (Pc), basal cell (Bc). Bar 20 µm and bar 10 µm. (**C**) Immunogold labeling with JIM8 in the pedestal cell; wall ingrowths (in). Bar 400 nm. (**D**) Arabinogalactan proteins (labeled with JIM8) were detected in the terminal cells of quadrifid. Bar 10 µm.

The epitope recognized by the JIM8 antibody was mainly detected in the pedestal cells. The strong fluorescence signal detected by JIM8 was observed in the cell wall ingrowths of the pedestal cell (Figure 4A,B). The gold particles were particularly numerous in the cell wall ingrowths (Figure 4C). In some quadrifids, the epitope recognized by the JIM8 antibody was also detected in the terminal cells in the cell walls of the arms (Figure 4D). Some signals in the form of dots were observed in the arm vacuoles (Figure 4D).

**Figure 5:**
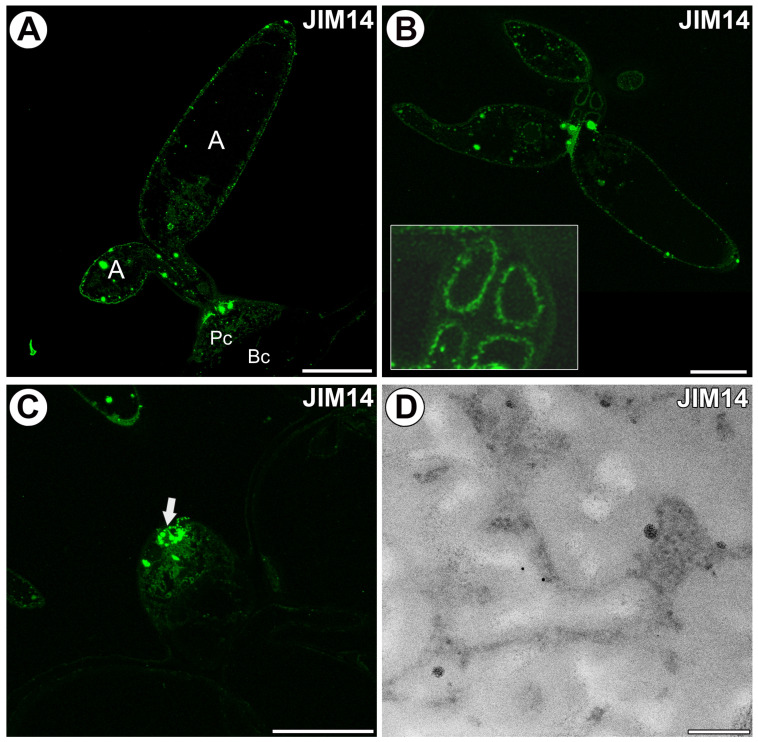
Arabinogalactan proteins detected in the quadrifids labeled with JIM14 (green color—signal of antibody). (**A**) Arabinogalactan proteins (labeled with JIM14) were detected in the quadrifid; arm (A), pedestal cell (Pc), basal cell (Bc). Bar 10 µm. (**B**) Arabinogalactan proteins (labeled with JIM14) were detected in the terminal cells. Note the intensive signal in the internal part of the cell wall near the protoplasts in the stalk (magnified fragment). Bar 10 µm. (**C**) Arabinogalactan proteins (labeled with JIM14) were detected in the pedestal cell, and a more intense signal in terminal cells (the basal part of terminal cells, arrow). Bar 20 µm. (**D**) Immunogold labeling with JIM8 in the pedestal cell; note only a few gold particles. Bar 200 nm.

The epitope recognized by the JIM14 antibody was observed in the cell walls of the arms of terminal cells (Figure 5A). The fluorescence signal detected by JIM14 was observed in the cell wall of the stalk of the terminal cell in the inner layer of the cell wall, near the protoplast (Figure 5B). A weak fluorescence signal detected by JIM14 was observed in the cell wall ingrowths of the pedestal cell (Figure 5C). Only some gold particles were localized in the cell wall ingrowths (Figure 5D).

**Figure 6:**
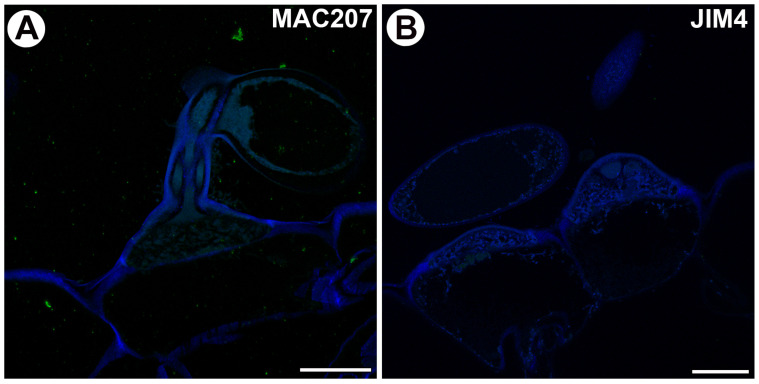
Arabinogalactan proteins detected in the internal trap glands labeled with MAC207 and JIM4. (**A**) Arabinogalactan proteins (labeled with MAC207) were detected in the quadrifid. Note that there is no positive signal. Bar 10 µm. (**B**) Arabinogalactan proteins (labeled with JIM4) were detected in the bifids; note that there is no positive signal. Bar 10 µm.

No fluorescence signals of the AGP epitope recognized by MAC207 and JIM4 were observed in the quadrifid or bifid cells (Figure 6A,B).

## 3. Discussion

Regarding the ultrastructure of quadrifids, our observations are consistent with the results presented by Fineran and Lee [11,30], who studied *Utricularia monanthos*. Currently, this taxon is treated as a subspecies of *Utricularia dichotoma* [31]. Apart from *U. dichotoma,* only a few species of *Utricularia* were investigated in the case ultrastructure of quadrifids: *Utricularia vulgaris* [32], *Utricularia* x *australis* [12,33,34,35,36], *Utricularia intermedia* [37], *Utricularia multifida*, and *Utricularia westonii* [38]. All of these authors found that the pedestal cell is a specialized transfer cell. It should be noted that while the basic architecture of quadrifids is constant in terms of the general shape of the arms and the size or number of these trichomes in the traps, there can be drastic differences between species [3]. Therefore, the shape of the quadrifids can help distinguish between some species [39]. However, it should be remembered that, within a population, a single individual, or even a trap, there can be differences in the shape of the trichomes (in the angle of the arms) [40,41]. Yang et al. [42] proposed that in the genus *Utricularia*, there is an evolutionary trend of reduction in the arm number (in the sense of reducing the number of terminal cells) of the internal trap’s trichomes, and the occurrence of quadrifids and bifids is an ancestral characteristic. However, internal trichomes with one terminal cell (monofids) were found in *U. multifida* and *U. westonii* [38]. Also, these monofids have pedestal cells that function as barrier and transfer cells. This indicates that the overall architecture of the inner trichomes from *Utricularia* traps is evolutionarily stable despite differences in the number of terminal cells.

When whole quadrifids were analyzed (without sectioning), it was only possible to detect AGPs in the arms [29]. We proposed that this was connected to the cuticle structure and the availability of cell wall components for antibodies. Fineran and Lee [11] described that the outermost region in the wall of the stalk in *Utricularia monanthos* (part of the terminal cell) is also heavily impregnated with an opaque cuticular material, and cuticular impregnation also occurs at the base of the arms. We obtained similar results in *Utricularia dichotoma* subsp. *novae-zelandiae* in the case of cell wall cutinization in quadrifid cells. In addition, we were also able to use not only TEM, but also SEM to demonstrate discontinuities in the cuticles in the middle and apical parts of the quadrifid arms. These cuticular discontinuities enable the uptake and transport of substances. Fineran and Gilbertson [43] demonstrated apoplastic pathways in *Utricularia* trichomes using lanthanum and uranyl salts as tracers. Juang et al. [44] showed the movement of the fluorescent symplastic tracer through the quadrifids of *Utricularia gibba*. In the sectioned material using the immunogold method, we found that in the arms, AGPs (labeled by JIM13) were present mainly in the inner part of the cell wall. This result is unsurprising because some AGPs are anchored to the cell membrane. Furthermore, AGPs are thought to be essential in establishing a plasma membrane–cell wall connection [17,45]. Of interest is the finding of AGPs in those parts of the cell wall of terminal cells, where cutin cystoliths are present. However, gold particles were mostly found between cutinized regions (in fibrillar, unimpregnated wall material). Also, in the stalk, AGPs occurred in the inner part of the cell wall. The outer part of the cell wall in the stalk was impregnated with cutin. We show that the terminal cells of quadrifids of *Utricularia dichotoma* subsp. *novae-zelandiae* are transfer cells because we observed cell wall ingrowths. In *Utricularia monanthos,* Fineran and Lee [11] described only short unbranched wall ingrowths in the arms. In contrast to these authors, we also observed branched wall ingrowths in the arms. This is related to the fact that the cell wall can be remodeled, and wall outgrowths are formed depending on the activity of the cell (transport intensity) [46]. We localized AGPs (labeled by JIM13) in wall ingrowths in the arms. We also demonstrated the presence of AGPs in the wall labyrinth in the pedestal cell. Previously, we could not locate AGPs in this cell because of the thick, heavily impregnated cell wall, which proved to be a barrier to antibodies [29]. It should be noted, however, that not all AGPs were found (MAC207, JIM4), indicating the variation in cell wall composition and the role of individual AGPs in different plants. According to Merced and Renzaglia [47], the modifications in cell wall architecture and composition may reflect taxonomic variability, functional differences, and environmental adaptations, as well as combinations of these. We found differences in the individual AGPs detected by the respective antibodies in the cell walls of quadrifid cells. AGPs labeled by JIM13 were the most widespread in the cell walls of quadrifid cells. It is thought that these AGPs may play various roles in plants, e.g., in embryo germination, cotyledon formation [48], in the positioning of cells during organ formation [49], in defense against pathogenic attack [50], and in abiotic stress responses such as salinity-modulating cell wall expansion [51], but also in sclerenchyma cells and xylem differentiation [52]. 

It should be highlighted that AGPs are prime mediators between the cell wall, the plasma membrane, and the cytoplasm [53]. AGPs also impact symplastic transport following changes in the plasmodesmata structure, acting through the regulation of cell wall properties surrounding the plasmodesmata [54]. For these reasons, the presence of AGPs in pedestal cells is not surprising, given their function. According to Fineran and Lee [11], all substances (nutrients from digested prey, water) absorbed by terminal cells must eventually pass symplastically through the pedestal cell if they are to be transported from the trap lumen into the walls of the trap. However, the presence of AGPs in the pedestal cell may be related to the fact that AGPs regulate the formation of wall ingrowths, as shown by Vaughn et al. [25] in *Vicia faba*. Because AGPs have a role in coordinating the required localized assembly of wall components, they play a role in forming cell wall ingrowths [46]. AGPs were found in cell wall ingrowths of various species [25,26,27,28,55,56]. AGPs were also found in cell wall ingrowths of various glands from traps of carnivorous plants from the genera *Aldrovanda* [57,58], *Dionaea* [59,60], and *Drosophyllum* [61,62]. According to Płachno and Kapusta [29], the presence of AGPs in the cell wall of terminal cells may be related to processes of endocytosis and exocytosis (involving vesicle trafficking and membrane recycling), in which these proteins participate [51,63,64]. However, the presence of AGPs in these cells may also be related to the presence of wall ingrowths. 

Yariv reagent is a synthetic aromatic glycoconjugate that can precipitate arabinogalactan proteins, namely, their ß-D-(1→3)-galactan structures [65]. Blocked AGPs with Yariv reagent cause a reduction in wall ingrowth density in the cotyledon epidermal transfer cells of *Vicia faba* [25]. Using this reagent may be key to testing whether perturbations in the development of cell wall ingrowths affect the functioning of traps in *Utricularia*.

## 4. Materials and Methods

### 4.1. Plant Material

*Utricularia dichotoma* subsp. *novae-zelandiae* (Hook.f) R.W. Jobson [31] plants were grown in the greenhouses of the Botanical Garden of Jagiellonian University. The plants were cultivated in wet peat under natural sunlight exposition.

### 4.2. Histological and Immunochemical Analysis

The traps were fixed in 8% (*w*/*v*) paraformaldehyde (PFA, Sigma-Aldrich, Sigma-Aldrich Sp. z o.o. Poznań, Poland) mixed with 0.25% (*v*/*v*) glutaraldehyde (GA, Sigma-Aldrich, Sigma-Aldrich Sp. z o.o. Poznań, Poland) in a PIPES buffer overnight at 4 °C. The PIPES buffer contained 50 mM PIPES (piperazine-N,N′-bis [2-ethanesulfonic acid], Sigma-Aldrich, Sigma-Aldrich Sp. z o.o. Poznań, Poland), 10 mM EGTA (ethylene glycol-bis[β-aminoethyl ether]N,N,N′,N′-tetraacetic acid, Sigma Aldrich, Poznań, Poland), and 1 mM MgCl_2_ (Sigma-Aldrich, Sigma-Aldrich Sp. z o.o. Poznań, Poland) at a pH of 6.8. For the analysis of the occurrence of the major cell wall polysaccharides and glycoproteins, the plant material was dehydrated with acetone and embedded in an Epoxy Embedding Medium Kit (Sigma Aldrich, Poznań, Poland). Ultrathin sections were cut on a Leica ultracut UCT ultramicrotome (Leica, Wetzlar, Germany). The rehydrated sections in PBS buffer were blocked with 1% bovine serum albumin (BSA, Sigma-Aldrich) in a PBS buffer and incubated with the following primary antibodies overnight at 4 °C: anti-AGP—JIM8, JIM13, JIM14, MAC207 and JIM4 [66]. All of the primary antibodies were used in a 1:20 dilution. They were purchased from Plant Probes, UK, and the goat anti-rat secondary antibody conjugated with FITC was purchased from Abcam (Cambridge, UK). The chromatin in the nuclei was stained with 7 µg/mL DAPI (Sigma-Aldrich) diluted in a PBS buffer. The samples were then cover-slipped using a Mowiol mounting medium: a mixture of Mowiol ^®^4-88 (Sigma-Aldrich) and glycerol for fluorescence microscopy (Merck, Warsaw, Poland) with the addition of 2.5% DABCO (Carl Roth GmbH + Co. KG, Karlsruhe, Germany). They were viewed using a Leica STELLARIS 5 WLL confocal microscope with lightning deconvolution. At least two different replications were performed for each of the analyzed traps, and about five to ten sections from each organ were analyzed for each antibody used. Negative controls were created by omitting the primary antibody step, which caused no fluorescence signal in any of the control frames for any stained slides (Figure S1). Semi-thin sections (0.9–1.0 µm thick) were prepared for light microscopy (LM) and stained for general histology using aqueous methylene blue/azure II (MB/AII) for 1–2 min.

### 4.3. Immunogold Labeling Distribution of AGP

A Leica Ultracut UCT ultramicrotome was used to prepare ultrathin sections (50 nm). They were blocked in 1% BSA (Aurion, Wageningen, The Netherlands) in PBS buffer for 15 min and then incubated in primary antibodies in a 1:10 dilution overnight at 4 °C. This was followed by washes in PBS buffer (6 × 5 min) and the samples were incubated with the goat anti-rat secondary antibody conjugated with 10 nm colloidal gold (Sigma Aldrich, Poland) in a 1:50 dilution for 2 h, followed by washing in PBS buffer and distilled water. Negative controls were created by omitting the primary antibody step. Lead citrate (Microshop, PIK Instruments Sp. z o.o., Piaseczno, Poland) and URANYLess (Microshop, PIK Instruments Sp. z o.o., Piaseczno, Poland) were added as contrasting agents. The cells were visualized using a Jeol JEM 100 SX microscope (JEOL, Tokyo, Japan) at 80 kV in the Department of Cell Biology and Imaging, Institute of Zoology, Jagiellonian University, Kraków, or a Hitachi UHR FE-SEM SU 8010 microscope (Hitachi, Tokyo, Japan) housed at the University of Silesia in Katowice.

### 4.4. Scanning Transmission Electron Microscopy

The glands were also examined using electron microscopy, as follows: Fragments of the traps were fixed in a mixture of 2.5% glutaraldehyde with 2.5% formaldehyde in a 0.05 M cacodylate buffer (Sigma-Aldrich, Sigma-Aldrich Sp. z o.o., Poznań, Poland; pH 7.2) for several days, and later, the material was processed as in Płachno et al. [67]. The material was dehydrated with acetone and embedded in an Epoxy Embedding Medium Kit (Fluka). Ultrathin sections were cut on a Leica ultracut UCT ultramicrotome. The sections were examined using a Hitachi UHR FE-SEM SU 8010 microscope housed at the University of Silesia in Katowice.

### 4.5. Scanning Electron Microscopy

For the scanning electron microscopy (SEM), the traps were fixed in a mixture of 2.5% glutaraldehyde with 2.5% formaldehyde in a 0.05 M cacodylate buffer and later washed in buffer and transferred to ethanol, and then transferred to acetone and dried using supercritical CO_2_. The material was then sputter-coated with gold and examined using a Hitachi S-4700 scanning electron microscope (Tokyo, Japan), which is housed at the Institute of Geological Sciences, Jagiellonian University, Kraków, Poland, or a Hitachi UHR FE-SEM SU 8010 microscope, which is housed at the University of Silesia in Katowice.

## 5. Conclusions

Our cytological study showed the presence of AGPs in the wall labyrinth in the pedestal cell, cell wall, and wall ingrowths of terminal cells. We found differences in the individual AGPs detected by the respective antibodies in the cell walls of quadrifid cells. AGPs labeled by JIM13 were the most widespread. Future studies using β-D-glucosyl Yariv reagent to block AGPs or using appropriate mutants will allow us to see how AGPs act on the development and functioning of not only the glands, but also the traps of *Utricularia*.

## Data Availability

The data presented in this study are available on request from the corresponding author.

## Supplementary Materials

The following supporting information can be downloaded at: https://www.mdpi.com/article/10.3390/ijms25126623/s1.

## Abbreviations

AGPs—arabinogalactan proteins, LM—light microscope, SEM—scanning electron microscope, STEM—scanning transmission electron microscopy.
