# Supplementary material for: Do Arabinogalactan Proteins Occur in the Transfer Cells of Utricularia dichotoma?
Source: Int J Mol Sci. 2024 Jun 16;25(12):6623. doi: 10.3390/ijms25126623 (PMC11204157; doi:10.3390/ijms25126623)

Figure S1

Figure S1. A. Control reactions of cell wall components after immunolabeling, section through the quadrifids, apparent lack of green, positive signal of AGPs presence, bar 10 µm.

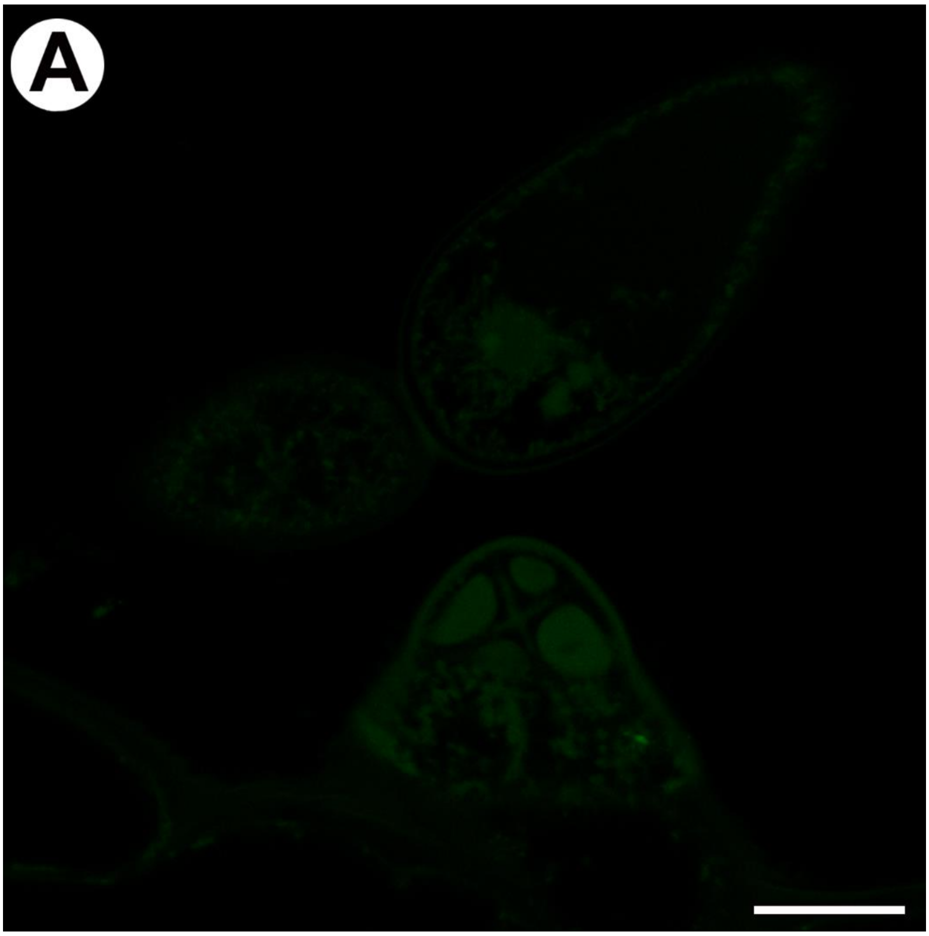

Supplement: Supplementary file 1 [file ijms-25-06623-s001.zip › ijms-3022586-supplementary.pdf]
